# Identifying Hub Genes and miRNAs Associated with Alzheimer’s Disease: A Bioinformatics Pathway to Novel Therapeutic Strategies

**DOI:** 10.3390/biom14121641

**Published:** 2024-12-20

**Authors:** Elisa Gascón, Ana Cristina Calvo, Nora Molina, Pilar Zaragoza, Rosario Osta

**Affiliations:** 1 Department of Anatomy, Embryology and Animal Genetics, University of Zaragoza, 50013 Zaragoza, Spain; egascon@unizar.es (E.G.); accalvo@unizar.es (A.C.C.); pilarzar@unizar.es (P.Z.); 2 Centro de Investigación Biomédica en Red de Enfermedades Neurodegenerativas (CIBERNED), Av. Monforte de Lemos 3-5, 28029 Madrid, Spain; 3 Agroalimentary Institute of Aragon (IA2), University of Zaragoza, 50013 Zaragoza, Spain; 4 Institute of Health Research of Aragon (IIS), Av. San Juan Bosco 13, 50009 Zaragoza, Spain

**Keywords:** bioinformatics, omics, neurodegenerative diseases (NDDs), extracellular/circulating biomarkers, microRNA, Alzheimer

## Abstract

Alzheimer’s disease (AD) is a neurodegenerative disorder that mainly affects the elderly population. It is characterized by cognitive impairment and dementia due to abnormal levels of amyloid beta peptide (Aβ) and axonal Tau protein in the brain. However, the complex underlying mechanisms affecting this disease are not yet known, and there is a lack of standardized biomarkers and therapeutic targets. Therefore, in this study, by means of bioinformatics analysis, AD-affected brain tissue was analyzed using the GSE138260 dataset, identifying 612 differentially expressed genes (DEGs). Functional analysis revealed 388 upregulated DEGs associated with sensory perception and 224 downregulated DEGs linked to the regulation and modulation of synaptic processes. Protein–protein interaction network analysis identified 20 hub genes. Furthermore, miRNA target gene networks revealed 1767 miRNAs linked to hub genes, among which hsa-mir-106a-5p, hsa-mir-17-5p, hsa-mir-26a-5p, hsa-mir-27a-3p and hsa-mir-34a-5p were the most relevant. This study presents novel biomarkers and therapeutic targets for AD by analyzing the information obtained with a comprehensive literature review, providing new potential targets to study their role in AD.

## 1. Introduction

Alzheimer’s disease (AD), initially characterized by Alois Alzheimer in 1907 [1], is pathologically marked by two defined events: the deposition of amyloid beta peptide (Aβ) in the intercellular space and the formation of intraneuronal tangles due to hyperphosphorylation of the axonal Tau protein [2]. The deposition of Aβ arises from the aberrant metabolism of amyloid precursor protein (APP), a transmembrane protein. In AD, dysregulation of APP cleavage leads to excessive accumulation of Aβ peptides. On the other hand, the tau protein, a microtubule-associated protein normally involved in maintaining neuronal stability, undergoes abnormal hyperphosphorylation in AD, promoting its aggregation into neurofibrillary tangles (NFTs). Key contributing mechanisms include aberrant phosphorylation and alterations in tau splicing, which disrupt its normal structure and enhance its pathological accumulation [3]. These pathological processes contribute to synaptic dysfunction and neuronal damage and ultimately lead to progressive neuronal death, which underlies the cognitive decline and neurodegeneration characteristic of AD. However, the first link that promotes these neurodegenerative processes remains unknown.

At present, AD is classified into familial or early-onset Alzheimer’s disease (EOAD), occurring before the age of 65, marked by mutations in genes transmitted in an autosomal dominant manner, and late-onset Alzheimer’s disease (LOAD), typically sporadic and occurring after the age of 65 [4]. EOAD represents less than 5% of all AD cases, with the majority attributed to LOAD. AD patients may present symptoms such as memory loss, language dysfunction, impaired visuospatial function, agnosia, and apraxia. Additionally, some individuals may experience anxiety and depression, among other neuropsychiatric symptoms [5,6]. In addition, environmental factors play a critical role in AD pathogenesis, such as aging, traumatic brain injuries, lifestyle habits, and chronic conditions like diabetes and hypertension significantly contribute to AD risk, hindering an earlier diagnosis and prognosis of the disease [3].

AD can be considered the predominant cause of dementia in older individuals, with prevalence doubling every six years of age, reaching 104.8 per 1000 person-years at 90 years or older [7]. This increasing concern establishes AD as the fifth leading cause of death in the world [2]. It is also suggested that up to 13.8 million AD patients may be diagnosed worldwide by 2050 [8], highlighting the need for research into effective diagnostic, preventive, and treatment strategies. Currently, AD diagnosis has traditionally been considered definitive only post-mortem. The clinical diagnosis consists of a detailed symptom history, neuropsychological assessment, and assessment of the impact of cognitive impairment on social, occupational, or other instrumental functions. As there are many underlying causes for dementia, the importance of accurate diagnostic biomarkers is underscored [7]. An ideal AD biomarker should identify key neuropathological features, demonstrating a diagnostic sensitivity and specificity exceeding 80% [8]. Existing biomarkers, such as Aβ 1-42 and tau protein levels in cerebrospinal fluid, ^18^F-FDG (18F-labeled fluorodeoxyglucose) in the cortex, are invasive or expensive and cause patient discomfort [5].

Advancements in whole genome sequencing, genomics, transcriptomics, proteomics, and bioinformatics provide substantial data for disease studies, such as finding new possibilities for potential diagnostic and prognostic targets in diseases or finding related genes and pathways between two or more different diseases, among others [6,9,10]. Bioinformatics methods provide applications in disease pathogenesis, molecular diagnostics, therapeutic drug targeting, and prognostic prediction. Relevant studies identified related genes in different AD stages [11] and therapeutic targets in AD brains through large-scale proteomic analysis [12]. Considering the complexity of this disease, these tools can enable the direct management of a high amount of data from diverse cohorts of AD patients and other neurodegenerative diseases, potentially aiding in the identification of specific biomarkers for AD. In this study, we analyzed the Gene Expression Omnibus (GEO) dataset GSE138260. The original authors of this database conducted a study to analyze significant evolutionary changes at the transcriptomic level. They also conducted expression studies, heatmap analyses, and systematic analyses at conserved sites to assess the evolution of the RNA profile [13]. The aim of this study is to introduce a novel approach utilizing the GSE138260 dataset to obtain new information relevant to late-onset Alzheimer’s disease (LOAD). The dataset exclusively represents LOAD samples, the most common form of AD. Firstly, differentially expressed genes (DEGs) were identified in AD samples versus healthy controls. Subsequent Gene Ontology (GO) and pathway enrichment analyses were performed to determine the functions and pathways affected by these DEGs. Furthermore, protein–protein interaction (PPI) networks were constructed to identify relevant subnetworks and hub genes. To make a step forward in this study, the potential role of micro RNAs (miRNAs) in AD was also investigated. miRNAs have emerged as critical regulators in the context of AD. Their ability to modulate multiple genes and their accessibility through non-invasive sampling, such as blood, position them as promising targets in complex diseases. Recent studies underscore their significance in AD by highlighting their influence on key pathogenic pathways. For instance, the dysregulation of miR-149 reduces BACE1 levels and serum Aβ accumulation, while the overexpression of miR-146a in microglia mitigates cognitive deficits, attenuates neuroinflammation, reduces Aβ levels, and prevents neuronal loss [3]. Accordingly, as a final step and novel contribution to this study, regulatory networks of miRNA target genes were constructed based on identified hub genes.

## 2. Materials and Methods

### 2.1. Dataset Selection

The Gene Expression Omnibus (GEO) was the selected database for this investigation. GEO is a globally accessible public repository established in 2000 for the investigation of gene expression datasets [14]. The search strategy used a combination of specific keywords: “Alzheimer disease” [MeSH Terms] OR Alzheimer [All Fields]. Rigorous cutoff criteria were implemented; the data search was confined to datasets exclusively associated with *Homo sapiens* and categorized under the study type of expression profiling by matrix. From the identified datasets, the GSE138260 dataset [13] was chosen for analysis. This dataset, hosted on the Agilent-034879 ADchip_1.0 platform, comprises a total of 36 samples (19 control samples and 17 AD samples). The target sample is brain tissue, specifically from the temporal cortex, a region prominently affected by Alzheimer’s disease [15]. Detailed information on these samples is available in Supplementary Table S1 (Table S1).

### 2.2. Study of Differentially Expressed Genes (DEGs)

The gene expression data were analyzed using RStudio version 4.4.1 environment and specific Bioconductor packages [16]: affy (v1.78.2) [17], oligo (v1.64.1) [18], GEOquery (v2.68.0) [19], limma (v3.56.2) [20], and ggplot2 (v3.4.3) [21]. Initial procedures included data correction and normalization using the Robust Multi-array Average (RMA) method implemented in the affy package. The RMA method applies background correction, quantile normalization, and probe summarization to ensure consistency and comparability of expression values across samples. Subsequently, the limma package’s moderated *t*-test, based on the empirical parametric Bayes method, facilitated the identification of differentially expressed genes (DEG) between Alzheimer’s disease (AD) patient and control samples. The criteria used for defining DEGs comprised |logFC| > 0.5 (1.4-fold change) for upregulated genes, |logFC| < −0.5 (0.7-fold change) for downregulated genes, and a *p*-value < 0.05. This cut-off criterion has been used in previous studies such as Cao et al. [22] and Yang et al. [23]. The discerned results were effectively visualized using a volcano plot generated with the ggplot2 package.

### 2.3. Functional and Enrichment Analysis of DEG Pathways

ClusterProfiler (v4.8.2) was the Bioconductor R package used for the functional enrichment analysis of up- and downregulated genes [24]. This package is a popular one renowned for its ability to perform comprehensive functional and pathway enrichment analyses, allowing for the analysis and visualization of enrichment across numerous organisms. The analysis specifically focused on Gene Ontology (GO) terms, categorizing them into (1) biological processes, (2) molecular functions, and (3) cellular components. Significance was determined with a stringent criterion: GO scores with a *p*-value < 0.05 were considered statistically significant. In this study, the functional enrichment analysis of up- and downregulated was analyzed with the predetermined statistical thresholds and the organism set to “org.Hs.eg.db”.

### 2.4. Constructing Networks of Protein–Protein Interactions (PPI) and Identifying Subnetworks

The STRING online database (https://string-db.org/) facilitated the prediction and analysis of protein–protein interactions up- and downregulated genes [25]. Cytoscape software (v3.9.1) was then used to visually represent these interactions, allowing for network modification and visualization [26]. Densely connected clusters within the networks were identified using the MCODE (Molecular Complex Detection) add-on of Cytoscape [27], applying specific criteria (degree limit = 2, node score limit = 0.2, kernel K = 2, max. depth = 100). The highest-scoring sub-networks for both positively and negatively regulated genes were subsequently selected. For further analysis and enrichment, Metascape (https://metascape.org) was used. Metascape is an intuitive online bioinformatics portal known for its user-friendly interface and robust functional enrichment and interactome analysis capabilities [28]. This ensured an accurate investigation of the studied biological processes.

### 2.5. Analyzing Hub Genes and Protein–Protein Interaction Networks

The Cytoscape add-on, cytoHubba, possesses the capability to conduct topological analysis through 11 methods, with the most frequently utilized being the degree, MCC (maximum clique centrality), and betweenness [29]. In our study, this add-on was employed to identify the top ten hub genes exhibiting the highest value of MCC within the protein–protein interaction (PPI) networks of both up- and downregulated genes.

### 2.6. Predicting Mirnas Through Hub Genes as Targets

The multiMiR R package (v2.4.0) (http://multimir.ucdenver.edu/) played a pivotal role as a bioinformatics tool for predicting target genes and miRNA pairs [30,31]. This robust package integrates a collection of microRNAs/targets from 15 distinct miRNA databases, including TarBase, miRTarBase, miRecords, DIANA-microT, ElMMo, MicroCosm, miRanda, miRDB, PicTar, PITA, TargetScan, TAM 2.0, miR2Disease, Pharmaco-miR VerSe and PhenomiR. In our study, this comprehensive resource was used to predict miRNAs specific to both up- and downregulated hub genes. The target gene-miRNA regulatory network identified was delineated and visually represented by using the R igraph (v2.1.1) and ggraph (v2.2.1) packages [32,33].

## 3. Results

### 3.1. Study of Differentially Expressed Genes (DEGs)

**Figure 1:**
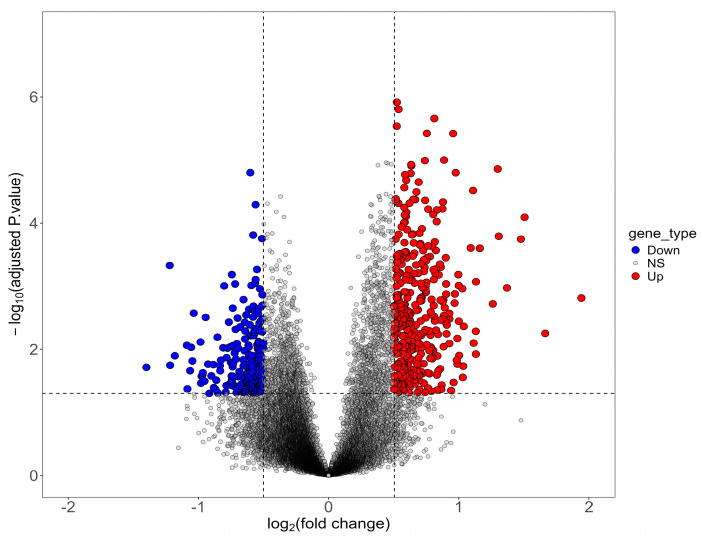
Volcano plot of differentially expressed genes (DEG) of GSE138260 dataset (LOAD’s and healthy control samples). Red dots represent upregulated genes according to *p*-value < 0.05 and |logFC| > 0. Blue dots represent downregulated genes according to *p*-values < 0.05 and |logFC| < 0.

The GSE138260 dataset contained a total of 22,564 genes, of which a total of 1876 differentially expressed genes (DEGs) were identified. Among these DEGs, there were 388 upregulated genes and 224 downregulated genes. The representation of these genes is illustrated in the volcano plot in Figure 1.

### 3.2. Functional and Enrichment Analysis of DEG Pathways

GO analysis consists of three parts: (1) biological processes (BP), (2) cellular component (CC), and (3) molecular function (MF). In this study, a functional analysis of upregulated DEGs and downregulated DEGs was performed (Figure S1).

The analysis revealed that upregulated DEGs were involved in biological processes such as sensory perception of chemical stimulus, detection of chemical stimulus, and detection of stimulus involved in sensory perception, among others. In terms of CC, DEGs were mainly enriched in intermediate filament, cornified envelope, intermediate filament cytoskeleton, and cell–cell junction. The molecular functions associated with these DEGs were those mainly related to olfactory receptor activity, odorant binding, and DNA-binding transcription activator activity.

On the other hand, downregulated DEGs were mainly involved in biological processes of modulation of chemical synaptic transmission, regulation of trans-synaptic signaling, and synaptic vesicle membrane organization. The most enriched CC terms for these DEGs are glutamatergic synapse, collagen-containing extracellular matrix, and external encapsulating structure. The molecular functions associated with these DEGs were glutamate receptor binding, polypeptide N-acetylgalactosaminyltransferase activity, and GTPase activity.

### 3.3. Analyzing Protein–Protein Interaction Networks

The construction of two protein–protein interaction networks was undertaken using STRING: one network for upregulated DEGs and another one for downregulated DEGs. The generated files were visualized through Cytoscape, and potential subnetworks were scrutinized using MCODE to explore the molecular networks in which these dysregulated genes might be implicated. A total of five subnetworks were identified for up- and downregulated DEGs. Among these sets, the subnetwork with the highest MCODE score was selected.

**Figure 2:**
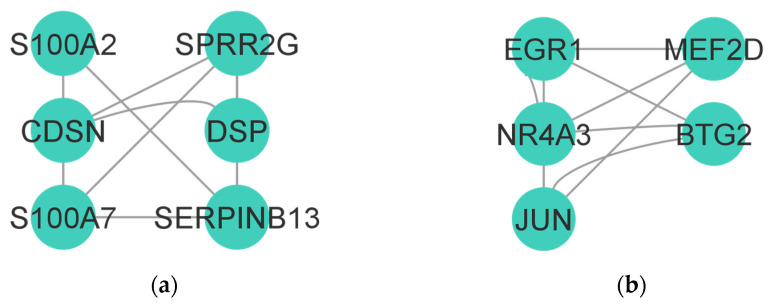
Analysis of differentially expressed gene (DEG) networks. (**a**) MCODE-clustered subnetwork of upregulated DEGs. (**b**) MCODE-clustered subnetwork of downregulated DEGs.

The main subnetwork of the upregulated DEGs consisted of six genes: *S100A2*, *CDSN*, *S100A7*, *SPRR2G*, *DSP*, and *SERPINB13* (Figure 2a). On the other hand, the main subnetwork of the downregulated DEGs consisted of five genes: *EGR1*, *NR4A3*, *JUN*, *MEF2D,* and *BTG2* (Figure 2b).

**Figure 3:**
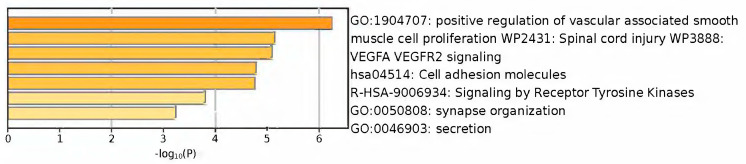
Enrichment analysis of MCODE-clustered subnetwork of upregulated DEGs by Metascape.

**Figure 4:**
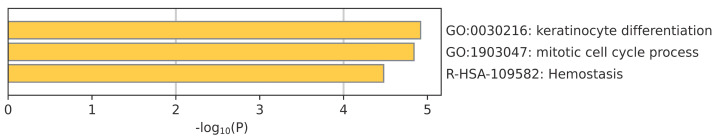
Enrichment analysis of MCODE-clustered subnetwork of downregulated DEGs by Metascape.

Lastly, these identified subnetworks underwent enrichment analysis using Metascape. Serving as a user-friendly online bioinformatics portal, Metascape expedites the accurate acquisition of functional enrichment, and interactome analysis results from a specified list of genes of interest. The upregulated DEGs subnetwork exhibited the most enriched pathways in positive regulation of vascular-associated smooth muscle cell proliferation, spinal cord injury, and VEGFA VEGFR2 signaling (Figure 3). In contrast, the downregulated DEGs subnetwork demonstrated the most enriched pathways in keratinocyte differentiation, mitotic cell cycle process, and hemostasis (Figure 4).

### 3.4. Analyzing Hub Genes

**Figure 5:**
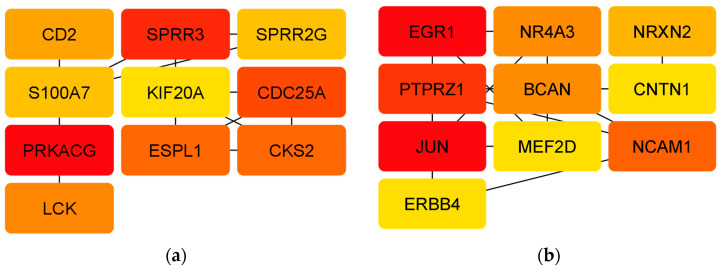
Hub genes identified by cytoHubba. (**a**) Hub genes of the PPI network of upregulated DEGs. (**b**) Hub genes of the PPI network of downregulated DEGs. The descending color from red to yellow represents decreasing interaction intensity between genes.

The 10 hub genes of the protein–protein interaction (PPI) network of upregulated DEGs with the highest MCC (maximum clique centrality) hub according to the cytoHubba complement were: *CD2*, *CDC25A*, *CKS2*, *ESPL1*, *KIF20A*, *LCK*, *PRKACG*, *S100A7*, *SPRR2G*, and *SPRR3* (Figure 5a). Conversely, the 10 hub genes in the PPI network of downregulated DEGs with the highest MCC hub were *PTPRZ1*, *NRXN2*, *NR4A3*, *NCAM1*, *MEF2D*, *JUN*, *ERBB4*, *EGR1*, *CNTN1*, and *BCAN* (Figure 5b).

The integration of information from 20 hub genes identified in the network analysis, along with subnetwork identification using MCODE, revealed a match in two genes (*S100A7* and *SPRR2G*) for upregulated DEGs and in four genes *(NR4A3*, *JUN*, *MEF2D*, and *EGR1*) for downregulated DEGs. This observation suggested that these hub genes might serve as promising biomarkers, potentially unveiling novel targets for Alzheimer therapeutics.

A literature review was conducted to determine whether research has been performed on each of these AD-related genes. Numerous studies have associated all hub genes identified from downregulated DEGs with Alzheimer’s disease (AD) and other neurological conditions like dementia and Parkinson’s disease, e.g., *NR4A3*, which is involved in the regulation of the immune system or *MEF2D,* which has a neuroprotective role [34,35,36,37,38,39,40,41,42]. On the other hand, *NRXN2* is considered a potential PD biomarker [43]. Conversely, for hub genes identified from upregulated DEGs, Alzheimer’s disease has been linked to *CD2*, *CDC25A*, *CKS2*, *LCK*, *PRKACG*, and *S100A7* [44,45,46,47,48,49]. In the case of *SPRR2G,* the gene is also connected to other neurological disorders such as dementia and Parkinson’s [50,51]. However, there is no available AD-related information for the *ESPL1*, *KIF20A*, *SPRR2G,* and *SPRR3* genes, which instead have associations with various cancers, including adenocarcinoma, medulloblastoma, tongue cancer, and pancreatic cancer, respectively [52,53,54,55].

### 3.5. Predicting Mirnas Through Hub Genes as Targets

A comprehensive analysis utilizing the multiMiR package, which integrates over 15 miRNA databases, revealed a total of 1767 microRNAs (miRNAs) associated with the hub genes. Specifically, 801 miRNAs were detected from hub genes of upregulated DEGs, while 966 miRNAs were detected from hub genes of downregulated DEGs. The detailed results are presented in Supplementary Tables S2 and S3 (Tables S2 and S3).

**Figure 6:**
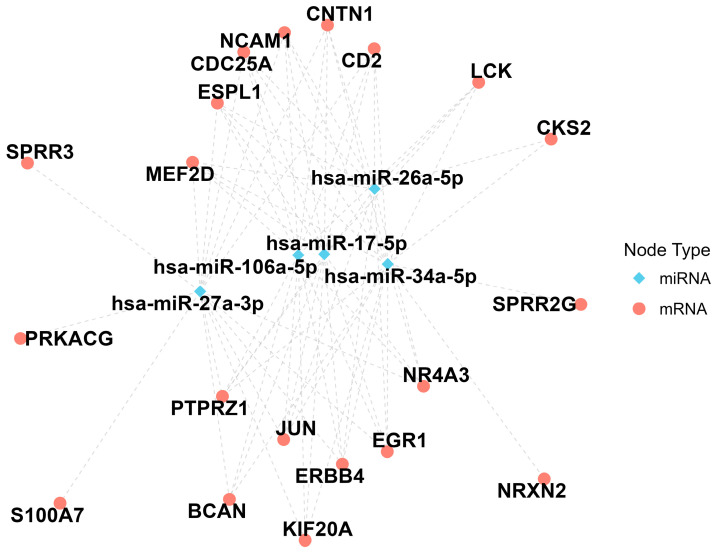
High centrality filtered network of miRNAs predicted from hub genes (mRNA). The blue diamond represents the miRNAs, and the red circle represents the mRNA. The dashed lines represent the relationships between them.

Given the extensive number of predicted miRNAs, a filtering process was implemented to select those targeting 10 or more hub genes and common to both upregulated and downregulated miRNAs. Subsequently, a miRNA-mRNA network centered on hub genes was constructed, and centrality measures (degree, betweenness, and closeness) were calculated to identify the top 5% of miRNAs occupying strategic positions in the network, potentially playing critical roles in Alzheimer’s disease (Figure 6). The miRNAs identified were hsa-miR-106a-5p, hsa-miR-17-5p, hsa-miR-26a-5p, hsa-miR-27a-3p, and hsa-miR-34a-5p. Lastly, an exhaustive bibliographic review was conducted to analyze the potential role of these miRNAs with Alzheimer’s disease, as in the case of has-miR-106a-5p [56].

## 4. Discussion

In the last few years, our understanding of the pathogenesis and development of Alzheimer’s disease (AD) has been improved. An increasing number of AD-related pathways and molecules are being identified, suggesting that multiple factors are involved in the onset and development of AD [57]. Although biomarkers based on tau or amyloid protein levels are currently available, many limitations still remain, making it necessary to search for new biomarkers [58]. Currently, many high-throughput computational approaches and high-throughput multi-omics technologies have been used for the identification of genes and pathways associated with Alzheimer’s disease. Interestingly, the study of multi-omics data (transcriptomics, proteomics, and metabolomics) aims to investigate new biomarkers and potential therapeutic targets for the study of the disease [59].

In this study, we provide a novel approach through new analyses additional to those conducted by the original authors of the GSE138260 database. We obtained 388 upregulated genes and 224 downregulated genes in differential gene expression analysis. The most significant pathways identified were related to the sensory perception of the upregulated genes and to the regulation and modulation of the synaptic process for the downregulated genes. This dysfunction has been shown to cause synaptic dysfunction, neuronal loss, and neurodegeneration. In particular, synapse loss is the anatomical factor best associated with cognitive deficits in AD patients [60,61]. In addition, several studies have linked impairment of sensory perception and hearing impairment with neurodegeneration and cognitive impairment [62,63]. These pathways align with the main symptom of Alzheimer’s disease, which is cognitive dysfunction [59].

For both up- and downregulated genes, hub genes with high biomarker potential were identified. The relevant upregulated hub genes were *CD2*, *CDC25A*, *CKS2*, *ESPL1*, *KIF20A*, *LCK*, *PRKACG*, *S100A7*, *SPRR2G* and *SPRR3.* No AD-related information is available for the *ESPL1*, *KIF20A*, *SPRR2G,* and *SPRR3* genes; however, they are associated with different types of cancer [52,53,54,55]. *CD2* is linked to CD2-associated protein (CD2AP), which is involved in the pathogenesis of sporadic AD. Polymorphisms in the *CD2AP* gene may induce its loss of function, correlating with increased Aβ deposition and tau pathology [44,64]. *CDC25A* has been identified as a critical factor in AD neurodegeneration. Specifically, it has been described that the inhibition of *CDC25A* exerted neuronal protection against nerve growth factor (NGF) deficiency and Aβ-induced cytotoxicity [45]. *CKS2*, part of the protein kinase family involved in cell cycle regulation, is linked to cell survival. Transcriptional modulation of *CKS2* may influence neuronal cell cycles, potentially mediating Aβ-dependent pathology in AD [46]. *LCK* is crucial in T-cell development and activation. It aids in phosphorylating ZAP70, a vital protein in T-cell pathways. Reduced ZAP70 levels result in immunodeficiency, a condition often impaired in AD [47]. *PRKACG* controls organelle dynamics by interacting with the anchoring protein A-kinase. Deletion of *PRKACG* results in a significant rise in Aβ secretion and may inhibit Gap junction channels, compromising the blood–brain barrier and leading to brain damage [48,65]. Lastly, *S100A7*, a calcium-binding protein, regulates processes associated with AD [49]. Its increased mRNA expression in the brain correlates with AD dementia and amyloid neuropathology. *S100A7* is thought to promote α-secretase activity, which prevents the generation of amyloidogenic peptides in AD [66].

Conversely, the relevant downregulated hub genes were *PTPRZ1*, *NRXN2*, *NR4A3*, *NCAM1*, *MEF2D*, *JUN*, *ERBB4*, *EGR1*, *CNTN1* and *BCAN*. *PTPRZ1*, linked to receptor-type tyrosine phosphatase zeta, is a potential susceptibility gene for schizophrenia and may affect working memory in mice [34,67]. It is involved in axonogenesis, myelination, and neuroinflammatory responses [67]. Thus, *PTPRZ1* may regulate cognitive and neuronal pathways, potentially influencing AD development [59]. *NRXN2*, involved in neuronal and synaptic functioning, is predominantly expressed in the substantia nigra, a region affected in Parkinson’s disease (PD), and is considered a potential PD biomarker [43]. Despite the distinct clinical and pathological characteristics of PD and AD, these diseases can share molecular mechanisms, and PD patients could also present an increased risk of developing AD dementia [68]. Hence, investigating *NRXN2* could reveal its role in AD. *NR4A3*, part of the *NR4A* gene family (which includes *NR4A1*, *NR4A2,* and *NR4A3*), is involved in CNS development and immune regulation, while *NR4A1* expression is notably decreased in AD patients. *NR4A3*’s role in long-term memory formation suggests it could be a therapeutic target for restoring cognitive function in AD using HDAC inhibitors and CRTC1 transcription regulation [35,69]. *NCAM1* (neuronal cell adhesion molecule 1) demonstrates the capability to decrease amyloid concentrations, consequently reducing its cytotoxic effects and ameliorating neuroinflammation associated with AD [36]. *MEFD2*, associated with autophagy [70], has been implicated in providing neuroprotection against various neurotoxins in neurological conditions like Alzheimer’s and Parkinson’s disease [37]. In this study, as well as in certain bioinformatic analyses related to dementia [50], *JUN* (also known as c-*JUN*), a proto-oncogene primarily associated with breast cancer, has been identified [71]. In the context of AD, this molecule has been implicated in promoting cell apoptosis and neurodegeneration in patients’ brains, suggesting its potential as a biomarker candidate [38]. The function of *ERBB4* in humans remains poorly understood to date. A recent study suggests that reduced *ERBB4* expression promotes heightened proliferation of human astrocytes, crucial for maintaining brain homeostasis and supporting neurons, particularly in AD [39]. *EGR1* plays a key role in memory formation and has been described to regulate the expression of genes involved in clathrin-mediated endocytosis, vesicular transport, and synaptic transmission, pathways that may be critical for AD pathogenesis [40,72]. *CNTN1* plays an important role in the neuroinflammatory process in AD. Overexpression of *CNTN1* in the hippocampus leads to adverse effects such as neuroinflammation, microglia activation, and cognitive dysfunction [41]. Finally, *BCAN* is a CNS-specific proteoglycan expressed in both astrocytes and neurons. Its potential nature as a biomarker in Alzheimer’s disease (AD) has been suggested and investigated, revealing notable differences in cerebrospinal fluid (CSF) samples [73].

Currently, miRNAs present new opportunities for disease investigation due to their regulatory functions and greater accessibility. One objective of this study is to link hub genes involved in AD pathology to more accessible epigenetic molecules, such as miRNAs. Using this novel approach, we conducted a comprehensive computational and bioinformatics analysis, identifying a network of 1767 miRNAs targeting these hub genes. After filtering and selecting the top 5% based on centrality measures, the most significant miRNAs were identified as hsa-miR-106a-5p, hsa-miR-17-5p, hsa-miR-26a-5p, hsa-miR-27a-3p, and hsa-miR-34a-5p. Downregulation of hsa-miR-106a-5p has been associated with a protective role in Alzheimer’s disease. It has been observed that overexpression of this miRNA reduces the levels of VEGFA, a factor known for its protective effects against cognitive decline in patients with AD [56,74]. Furthermore, other studies have identified this miRNA as a target of genes involved in fundamental processes such as axon guidance, immune responses, and actin cytoskeleton regulation, including p21-activated kinases. Axon guidance is a critical process for the formation of neural networks, and it has been demonstrated to be altered in conditions such as ALS and Parkinson’s disease [56,75]. Therefore, it is plausible that altered hsa-miR-106a-5p plays a crucial role in the pathophysiology of AD. Hsa-miR-17-5p appears to play a critical role in the pathogenesis of Alzheimer’s disease. Elevated levels of this miRNA in microglia surrounding Aβ deposits in the brains of AD patients suggest that it may mediate inflammatory responses in these environments [76]. Additionally, it has been shown to regulate essential genes, such as *F3* and *MKNK2*, possibly through competitive interactions with the lncRNAs LINC00365 and FBXL19-AS1 [77]. Hsa-miR-17-5p also targets TNF-α, a proinflammatory cytokine involved in the chronic neuroinflammation characteristic of AD, underscoring its significance in disease progression [78]. Its involvement in pathways associated with oxidative stress and metal-induced toxicity further highlights its relevance in neurodegenerative processes [79]. Therefore, hsa-miR-17-5p presents substantial potential as a target for novel therapeutic strategies in AD. Hsa-miR-26a-5p appears to play a significant role in cognitive impairment and the pathogenesis of AD by regulating the Wnt/β-catenin signaling pathway. Sadlon and coworkers reported that this miRNA targets a substantial proportion of brain-expressed genes (48.58%) and that its downregulation in individuals with cognitive deficits is associated with decreased activity in the Wnt/β-catenin pathway [80]. Reduced levels of Wnt/β-catenin have been specifically linked to increased Aβ deposition and cognitive decline in AD models, suggesting that the loss of hsa-miR-26a-5p may exacerbate these effects by impairing pathway activity [80,81]. Furthermore, this pathway is a known target of statins, a class of drugs with potential protective effects in AD [80,82]. Therefore, hsa-miR-26a-5p can be a promising target for future therapeutic interventions, warranting further investigation into the relationship between has-miR-26a-5p and the Wnt/β-catenin system in AD. Hsa-miR-27a-3p appears to play a complex role in AD, particularly in patients with comorbid depression, where its expression is further elevated. This miRNA may influence AD pathology by reducing Aβ clearance through the downregulation of FGF2 [83] and by interacting with critical genes associated with neurodegeneration and protein aggregation, including *BACE1*, *GSK3β*, *MAPT*, and *PSEN1*. Interestingly, lower levels of hsa-miR-27a-3p have been correlated with higher concentrations of tau biomarkers (T-tau and P-tau) in cerebrospinal fluid, suggesting a possible inverse relationship with tau pathology [84]. These findings indicate that precise regulation of hsa-miR-27a-3p could be essential for maintaining a balance between Aβ and tau accumulation. Further studies are warranted to determine whether targeted modulation of miR-27a-3p might provide therapeutic benefits in AD. Lastly, overexpression of hsa-miR-34a-5p has been shown to have negative effects on synaptic plasticity and neuronal survival. This miRNA inhibits genes critical for synaptic plasticity and neuronal metabolism, such as mitochondrial oxidative phosphorylation and the pentose phosphate pathway, both of which are essential for long-term neuronal survival [85,86]. Furthermore, the upregulation of hsa-miR-34a-5p in AD patients and its influence on apoptotic pathways underscore its role in neurodegenerative processes [87]. Additionally, hsa-miR-34a-5p has been observed to interact significantly with mixtures of B vitamins (B1, B2, B3, and B9), which are associated with improved cognitive performance. This suggests that these vitamins may modulate has-miR-34a-5p levels and counteract its detrimental effects in AD [86]. Collectively, these findings imply that this miRNA may play a key role in the pathogenesis of AD and thus could be proposed and further studied as a potential biomarker or therapeutic target.

Overall, all this information suggests that these targets may have a direct or indirect role related to neuroinflammation and other critical AD pathways, making them interesting as potential diagnostic and/or therapeutic biomarkers.

This study represents an initial evaluation through bioinformatics analysis, using a single gene expression profile comprising 36 brain samples from Alzheimer’s patients. Our findings provide valuable information on potential key molecular targets and pathways involved in Alzheimer’s disease. Some of these targets have already been shown and experimentally validated to play a key role in the disease, while others are promising candidates for future studies to further investigate their role in the mechanisms of Alzheimer’s disease. These validations will provide a better understanding of the progression and pathology of Alzheimer’s disease.

## 5. Conclusions

This study provides an opportunity to conduct an exploratory investigation using a significant number of brain samples to identify potential hub genes and miRNAs relevant to Alzheimer’s disease and analyze their roles using bioinformatics tools. The proposed candidate genes and miRNAs, which may serve as biomarkers and/or therapeutic targets, are mainly related to neuroinflammation, and they could play a critical role in the progression of Alzheimer’s disease. Taken together, these findings could enable a better understanding of the underlying mechanisms of the disease, and they might pave the way to innovative therapeutic strategies, offering new perspectives for the effective treatment and diagnosis of Alzheimer’s disease.

## Data Availability

Data used in this study are publicly available on the gene expression omnibus (GEO) database and can be accessed through GSE138260.

## Supplementary Materials

The following supporting information can be downloaded at: https://www.mdpi.com/article/10.3390/biom14121641/s1, Table S1: Table of GSE138260 Sample Set Information; Figure S1: Figure of Gene Ontology (GO) function and pathway enrichment analysis of upregulated DEGs and downregulated DEGs; Table S2: Table of miRNAs and their upregulated hub genes; Table S3: Table of miRNAs and their downregulated hub genes.
